# Inhibition of microbially mediated total organic carbon decomposition in different types of cadmium contaminated soils with wheat straw addition

**DOI:** 10.1038/s41598-024-64267-2

**Published:** 2024-07-02

**Authors:** Chengjuan Li, Hui Wang, Yajun Yang, Hexiang Liu, Xianhui Fang, Yaohui Zhang, Jialong Lv

**Affiliations:** 1 State Key Laboratory of Soil Erosion and Dryland Farming on the Loess Plateau, Institute of Soil and Water Conservation Chinese Academy of Sciences & College of Natural Resources and Environment, Northwest A&F University, Yangling, 712100 Shaanxi People’s Republic of China; 2 Xianyang Soil and Fertilizer Workstation, Xianyang, 712000 Shaanxi People’s Republic of China

**Keywords:** Cd-contaminated soils, Cumulative C mineralization, Wheat straw, Bacterial abundance, Soil organic carbon, Metals, RNA, Environmental sciences

## Abstract

Wheat straw returning is a common agronomic measure in the farmland. Understanding organic carbon transformation is of great significance for carbon budget under the premise of widespread distribution of cadmium (Cd) contaminated soils. An incubation experiment was conducted to assess the influence of Cd contamination on the decomposition and accumulation of total organic carbon (TOC) as well as the composition and abundance of bacterial communities in eight soil types with wheat straw addition. The results showed that inhibition of Cd contamination on microbially mediated organic carbon decomposition was affected by soil types. The lower cumulative C mineralization and higher TOC content could be observed in the acidic soils relative to that in the alkaline soils. The content of Cd in soil exhibits different effects on the inhibition in decomposition of TOC. The high dosage level of Cd had stronger inhibitory impact due to its high toxicity. The decomposition of TOC was restricted by a reduction in soil bacterial abundance and weakening of bacterial activities. Redundancy analysis (RDA) indicated that *Proteobacteria* and *Gemmatimonadetes* were abundant in alkaline Cd-contaminated soils with wheat straw addition, while *Bacteroidetes* dominated cumulative C mineralization in acidic Cd-contamination soils. Moreover, the abundance of predicted functional bacteria indicated that high-dose Cd-contamination and acid environment all inhibited the decomposition of TOC. The present study suggested that pH played an important role on carbon dynamics in the Cd-contaminated soils with wheat straw addition.

## Introduction

At present, wheat straw returning is widely applied in the main wheat producing areas in China. Wheat straw returning affects total organic carbon (TOC) mineralization and decomposition, which is of great significance for improving the cycling of various substances in soil^1,2^. TOC is predominantly decomposed under the action of microorganisms^3,4^. Therefore, investigating the dynamics in microbial communities is very important to understand the mechanism of carbon transformation in farmland soil with wheat straw returning.

Increasing evidence of the uncertainty of straw returning in heavy metal contaminated soil is a major concern. The heavy metal contamination of farmland is universal and widespread^5,6^. A nationwide soil survey showed that cadmium (Cd) was the most frequent contaminated trace metal in the investigated sites based on the Environmental Quality Standard for soils (GB15618-1995). The survey indicated that approximately 1.3 × 10^7^ hm^2^ of farmland in 25 areas of 11 provinces in China contaminated with Cd^7^. Previous researches have demonstrated that Cd contamination could affect microbial activities and thus influencing microbially mediated organic carbon decomposition in soil^8–11^. Therefore, understanding the impact of Cd pollution on organic carbon mineralization is of great significance for estimating global carbon budget in contaminated soils with straw returning.

The influence of Cd on TOC decomposition was closely associated with the types and natures of organic amendments^12,13^. Many studies focus on the effects of Cd on the decomposition and transformation of TOC after adding a variety of organic materials, such as animal manures, crop straws and composts^14,15^. For example, Darma et al. found that maize straw incorporation enhances the TOC decomposition and dissolved organic carbon concentration in the As and Cd contaminated soils^16^. Meanwhile, the dosage of Cd significantly affects the response of soil microorganisms and the organic carbon decomposition process. Generally, low concentration of Cd stimulates cumulative C mineralization and carbon metabolism, thus increasing organic carbon mineralization rate in soil. However, high Cd concentration can inhibit microbial decomposition activities, leading to a decrease in TOC mineralization rate^17^. However, previous researches suggested that Cd-contamination influences the decomposition of organic carbon in several types of soils^18,19^. Actually, the impact of Cd pollution on TOC decomposition is largely regulated by diverse soil properties especially soil pH. Although we are aware of the differences in the availability of Cd in acidic and alkaline soils with straw returning. It inevitably affects the abundance of microbial communities due to the various tolerance of microorganisms to acidity and alkalinity^20^. However, the microbial communities driving organic carbon decomposition in acidic and alkaline soils with straw returning under Cd stress are still unclear.

The objective of this study was to examine the inhibitory impacts of Cd on microbially mediated organic carbon decomposition in acidic and alkaline soils in a laboratory experiment. The laboratory incubation experiment is a well-controlled condition to understand the mechanism of the inhibitory effects on organic carbon decomposition and storage in heavy metal contaminated soils. Therefore, this study aims to explore: (1) the effect of Cd on the TOC transformation under straw returning; (2) the effect of Cd on the changes in microorganisms related to carbon decomposition; (3) the effect of Cd on the variations in abundances of carbon metabolism functional genes. This is of great significance for understanding the carbon budget in Cd-contaminated areas with wheat straw returning.

## Materials and methods

### Soil and wheat straw used for incubation

Soil samples were collected from agricultural soils (0–20 cm) in the main wheat producing area in eight provinces (Yunnan, Jiangxi, Gansu, Jilin, Henan, Inner Mongolia, Tianjin and Chongqing). The selected physicochemical properties are provided in Supplemental materials. According to the difference in soil pH, Yunnan, Jiangxi, Jilin and Chongqing are classified as acidic soils, while Henan, Gansu, Inner Mongolia and Tianjin are considered as alkaline soils. The soils were air-dried and sieved through a 2 mm mesh for incubation. Tested soils were contaminated with CdCl_2_ solution and then mixed thoroughly to reach 0 mg Cd kg^−1^, 1 mg Cd kg^−1^, 5 mg Cd kg^−1^ and 10 mg Cd kg^−1^, respectively. The soil samples were aged lasting for six months at 25 ± 2 ℃ in dark. During the aging, 70% field water holding capacity was maintained with distilled water added every three days. The Cd-contaminated soil samples obtained were used to conduct this incubation experiment. Wheat straw (WS) was acquired from no-contamination field in Yangling, Shaanxi Province, China and sieved less than 1 mm. Total carbon of wheat straw was 406.6 ± 2.13 g kg^−1^, total nitrogen was 8.06 ± 0.14 g kg^−1^, C/N was 50.42, pH was 6.52 ± 0.05.

### Incubation experiment design

A total of 500 g Cd contaminated soil was weighed and placed in a 1000 ml plastic bottle. In each treatment, 5% WS was added and thoroughly mixed with the soils. The treatments in this study were: 0 mg kg^−1^ Cd-contaminated soil with 5% WS addition served as the control (CK), 1 mg kg^−1^ Cd-contaminated soil with 5% WS addition (LW), 5 mg kg^−1^ Cd contaminated-soil with 5% WS addition (MW) and 10 mg kg^−1^ Cd contaminated-soil with 5% WS addition (HW). 32 treatments with triplicates were incubated at 25 ± 2 ℃ in the dark and performed in a 77-day incubation^21^. The moisture was kept at 70% water holding capacity through adding deionized water during the incubation. The bottles were opened to allow air circulation at each day for adding deionized water.

### Determination methods

Cumulative C mineralization was determined on the day of 1, 2, 3, 4, 5, 6, 7, 11, 14 and 77 during the incubation. 20 ml NaOH (0.5 mol L^−1^) was contained in a small cup for absorbing CO_2_ and placed in each plastic bottle including the CK treatment. The bottle was taken out on each specific measurement date and then titrated with 0.5 mol L^−1^ HCL^20,22^. The calculation of CO_2_ emission was referenced as the previous research^22^. On day 77, soil samples from each bottle were used to determine the soil physical and chemical properties. A part of the samples left to dry to the air and then pulverized and sieved through mesh, after which it was analyzed for its TOC and total nitrogen (TN) using a CN analyzer (Vario EL III Elementar, Germany). Soil pH was determined with a pH meter (pH7110, WTW, Germany). Cation exchange capacity (CEC) was measured applying ammonium acetate at pH 7.0. Calcium carbonate (CaCO_3_) concentration was measured by the gasometric method and clay content was determined introducing the standard pipet method. Available potassium (AK) was determined using flame photometry (6400A, INESA, China). Available phosphorus (AP) was measured using a spectrophotometer (UV-1700PC). Total Cd concentration was measured using inductively coupled plasma mass spectrometer (ICP-OES) according to standard soil testing method^23^. Another part of the samples were stored at—20 ℃ for the determination of 16S rRNA. The DNA samples were extracted, checked and quantified. On the IlluminaMiSeq PE300 sequencing platform, the bacterial 16S rRNA in the V3–V4 region was amplified and determined using the primers 338F and 806R. The related genes were predicted by the PICRUSt (Phylogenetic Investigation of Communities by Reconstruction of Unobserved States).

### Statistical analysis

Figures were performed by Origin 2016. Redundancy analysis (RDA) between cumulative C mineralization, TOC concentration and the abundance of dominant carbon-relating bacteria in different types of soil was performed using CANOCO 5.0. Correlation analysis was conducted by SPSS 23.0. Duncan’s multiple range tests were employed to examine differences between values. Differences in the values with P < 0.05 were considered statistically significant. The unweighted pair group method with arithmetic mean was applied for understanding the discrepancies of the whole bacterial community structure among all soils.

### Ethics approval and consent to participate

The authors declare that they have no known competing financial interests or personal relationships that seem to affect the work reported in this article. We declare that we have no human participants, human data, or human tissues.

## Results

### TOC concentration

**Figure 1:**
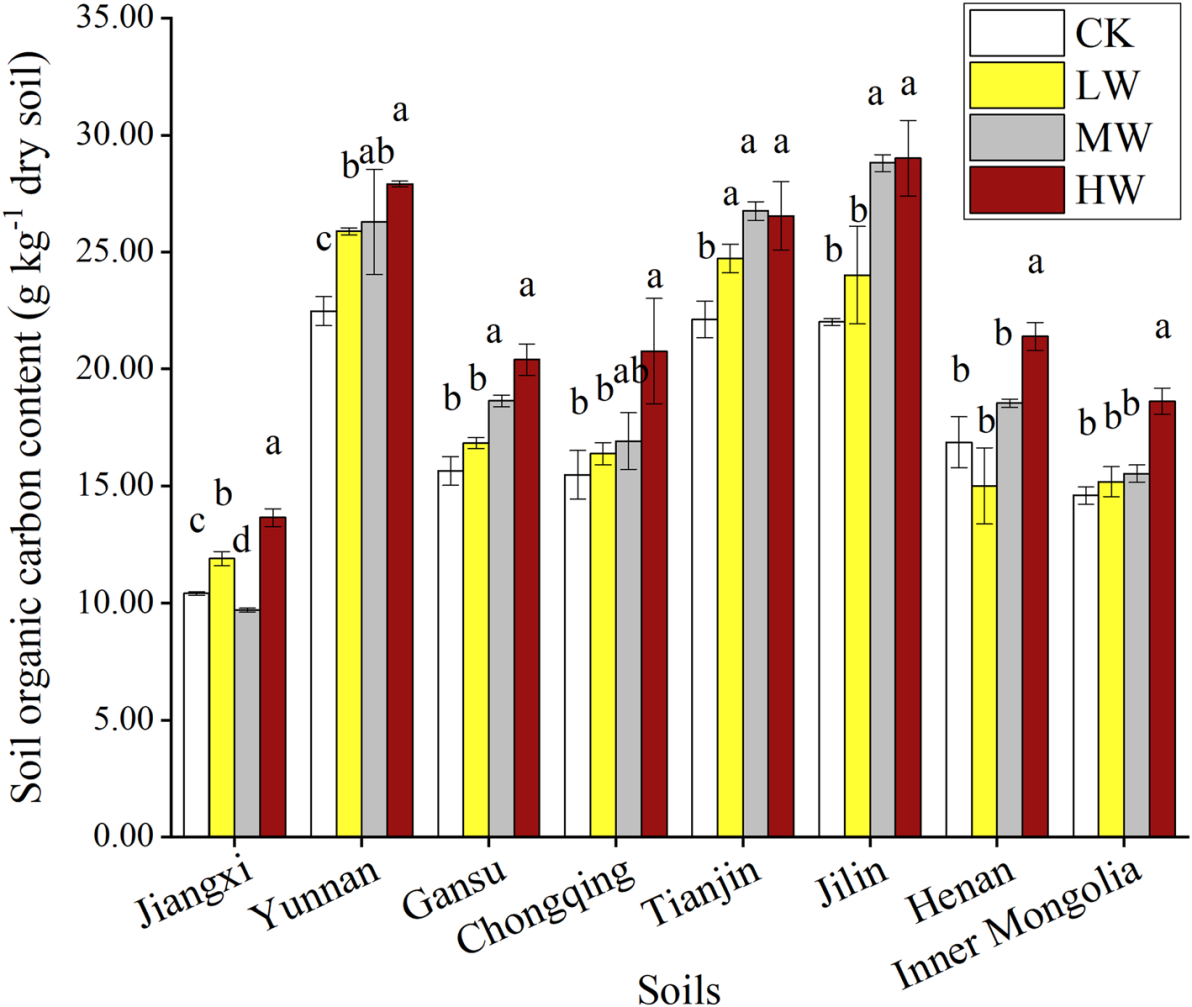
Soil organic matter content after the incubation with different dosages of Cd-contamination and wheat straw addition. (CK: 0 mg kg^−1^ Cd-contaminated-soil with 5% WS addition; LW: 1 mg kg^−1^ Cd-contaminated-soil with 5% WS addition; MW: 5 mg kg^−1^ Cd contaminated-soil with 5% WS addition; HW: 10 mg kg^−1^ Cd contaminated-soil with 5% WS addition).

The concentration of TOC in different types of soil with Cd-contamination and WS addition is shown in Fig. 1. The TOC content was with a range from 9.70 to 29.01 g kg^−1^ among all treatments. After the incubation, the TOC content was higher in all dosages of Cd-contaminated soils than that in the CK soils (except in MW-Jiangxi and LW-Henan soils). Compared with the CK treatments, the HW treatment significantly increased the TOC content by 24.20–34.16% in all soils (P < 0.05). The LW and MW treatment enhanced the TOC concentration by 4.12–15.21% and 6.45–30.88% (except in MW-Jiangxi and LW-Henan soils), respectively. However, there were significant changes between LW-Jiangxi and CK-Jiangxi, LW-Yunnan, MW-Yunnan and CK-Yunnan, LW-Tianjin, MW-Tianjin and CK-Tianjin, respectively. In addition, the average TOC content in the acidic soils with different dosages of Cd-contamination (20.10%) was higher than that in the alkaline soils (19.40%). In the acidic soils, the highest average TOC content was observed in the Jilin soils (22.01–29.01 g kg^−1^) and its lowest content was in the Jiangxi soils (9.70–13.65 g kg^−1^). In the alkaline soils, the maximum average TOC content in the Tianjin (22.12–26.76 g kg^−1^) and the minimum average content in the Inner Mongolia soils (14.59–18.62 g kg^−1^) could be observed.

### Cumulative C mineralization

**Figure 2:**
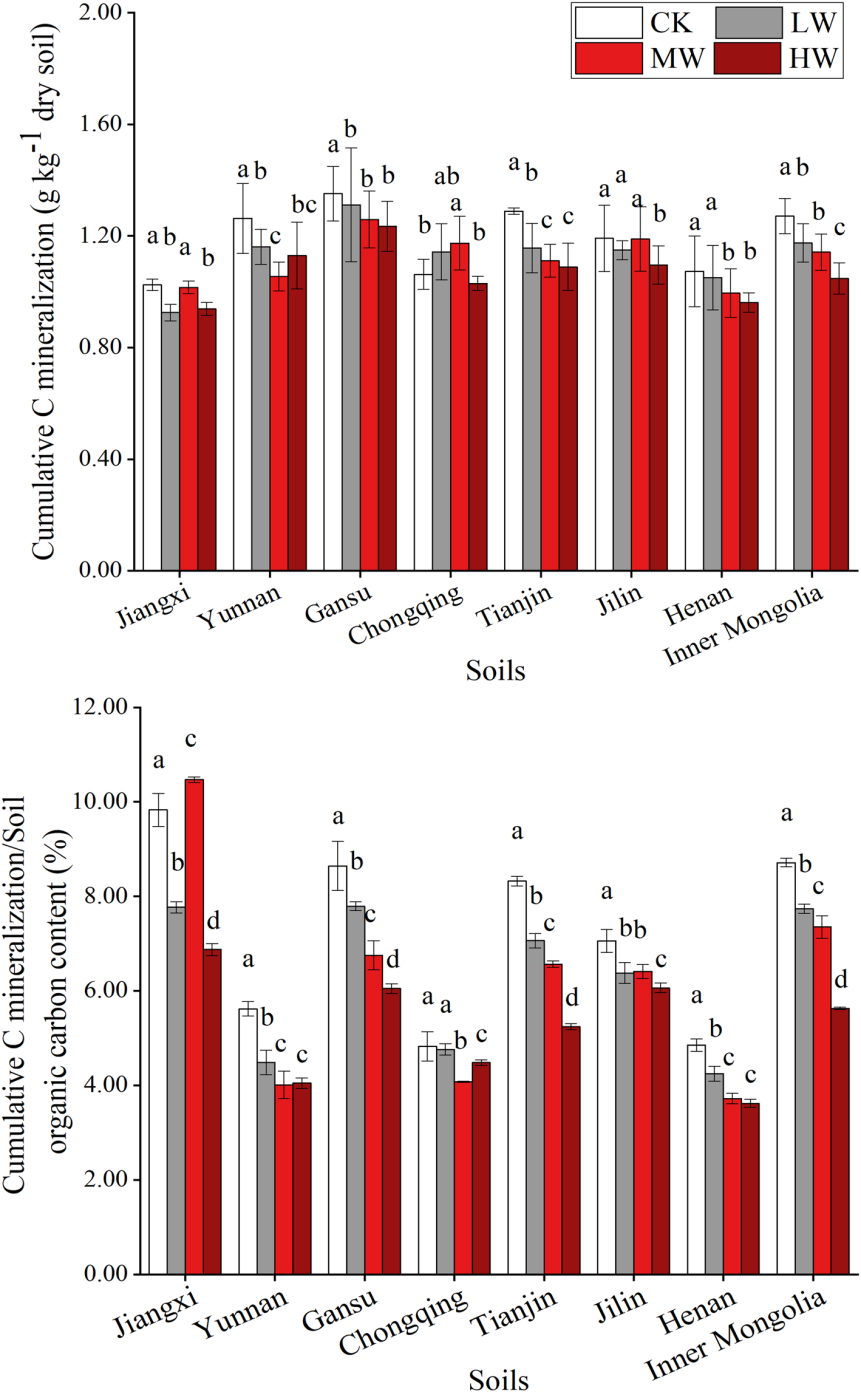
Soil respiration (**A**) and the percentage of soil respiration to soil organic carbon content (**B**) after the incubation with different dosages of Cd-contamination and wheat straw addition. (CK: 0 mg kg^−1^ Cd-contaminated soil with 5% WS addition; LW: 1 mg kg^−1^ Cd-contaminated soil with 5% WS addition; MW: 5 mg kg^−1^ Cd contaminated-soil with 5% WS addition HW: 10 mg kg^−1^ Cd contaminated-soil with 5% WS addition).

A similar decreasing trend for cumulative C mineralization along with the increasing dosages of Cd-contamination in all types of soils is shown in Fig. 2A. The content of cumulative C mineralization was higher in the CK treatment (averagely 1.19 g kg^−1^ in all soils) compared with that under the different dosages of Cd-contaminated treatments. The average cumulative C mineralization under the LW, MW and HW treatment in all soils was 1.13 g kg^−1^, 1.11 g kg^−1^ and 1.06 g kg^−1^, respectively. Relative to the CK treatment, the HW treatment significantly decreased the average cumulative C mineralization among all types of soils (P < 0.05; except in Chongqing soil). However, there were no regularly significant differences between LW, MW and CK treatment. After the incubation with different dosages of Cd-contamination, the average cumulative C mineralization in the alkaline soils were higher than that in the acidic soils. In different types of soils, the highest decreased rate of cumulative C mineralization in the Inner Mongolia soils and the lowest value in the Chongqing soils were observed. The percentage of cumulative C mineralization to TOC content in all soils showed a similar trend (Fig. 2B). It can be seen that percentage decreased along with the increasing of Cd-contamination level (except in MW-Jiangxi and HW-Chongqing soils). Furthermore, the average percentage value in the alkaline soils was higher than that in the acidic soils.

### Soil bacterial 16S rRNA gene abundances

**Figure 3:**
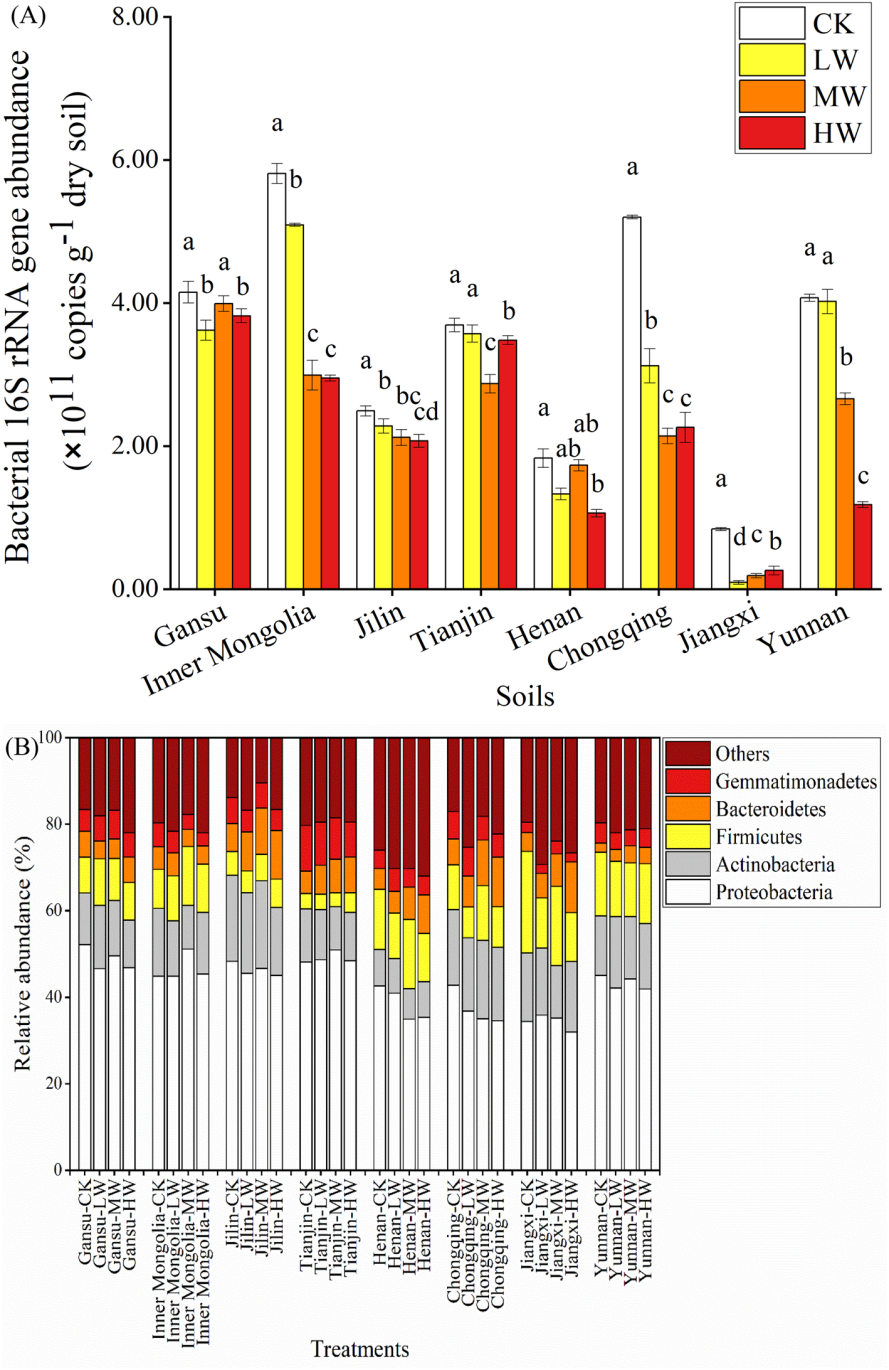
Soil bacterial 16S rRNA gene abundance (**A**) and structure (**B**) after the incubation with different dosages of Cd-contamination and wheat straw addition. (CK: 0 mg kg^−1^ Cd-contaminated soil with 5% WS addition; LW: 1 mg kg^−1^ Cd-contaminated soil with 5% WS addition; MW: 5 mg kg^−1^ Cd contaminated-soil with 5% WS addition HW: 10 mg kg^−1^ Cd contaminated-soil with 5% WS addition).

The effects of different dosages of Cd contamination and wheat straw addition on the changes of bacterial 16S rRNA gene abundance are shown in Fig. 3A. Compared with CK treatment, different dosages of Cd contamination together with WS addition reduced the abundance of soil bacterial 16S rRNA gene. After the incubation, the abundances of bacterial 16S rRNA gene in the CK treatments were within 8.39 × 10^10^ copies g^−1^ soil ~ 5.80 × 10^11^ copies g^−1^ soil, and the gene abundances in the LW, MW and HW treatment were 9.15 × 10^9^ copies g^−1^ soil ~ 5.09 × 10^11^ copies g^−1^ soil, 1.87 × 10^10^ copies g^−1^ soil ~ 3.99 × 10^11^ copies g^−1^ soil, and 2.61 × 10^10^ copies g^−1^ soil ~ 3.82 × 10^11^ copies g^−1^ soil, respectively. The largest decreased rates were observed in 10 mg kg^−1^ Cd contaminated-soils amended with 5% WS addition (except in LW-Gansu, MW-Tianjin, MW-Chongqing and LW-Jiangxi soils). Additionally, soil bacterial 16S rRNA gene abundances in the alkaline soils (3.25 × 10^11^ copies g^−1^ soil) were higher than that in the acidic soils (2.19 × 10^11^ copies g^−1^ soil). Correlation analysis revealed that there were significantly positive correlations between bacterial 16S rRNA gene abundances and cumulative C mineralization in all soils (r = 0.628, P < 0.01) (Fig. 5B). However, after analyzing the relationship in the alkaline soils and acidic soils alone, 16S rRNA gene abundance was significantly correlated with cumulative C mineralization in the alkaline soils (r = 0.768, P < 0.01), while it had no significant association with cumulative C mineralization in the acidic soils (r = 0.502, P < 0.05).

### Soil bacterial community structure

**Figure 4:**
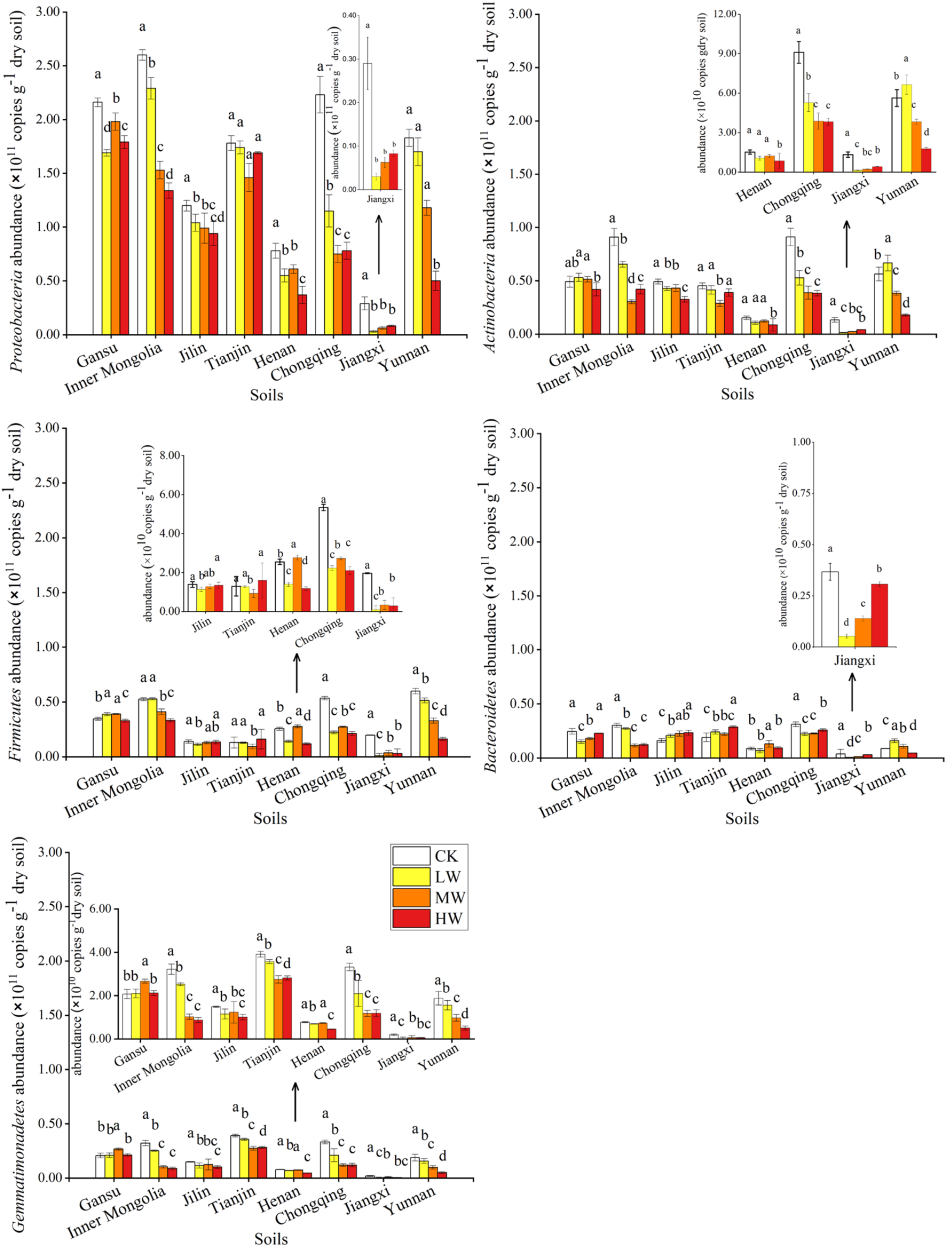
The abundance in the dominant carbon-relating phyla after the incubation. (CK: 0 mg kg^−1^ Cd-contaminated soil with 5% WS addition; LW: 1 mg kg^−1^ Cd-contaminated soil with 5% WS addition; MW: 5 mg kg^−1^ Cd contaminated-soil with 5% WS addition HW: 10 mg kg^−1^ Cd contaminated-soil with 5% WS addition).

**Figure 5:**
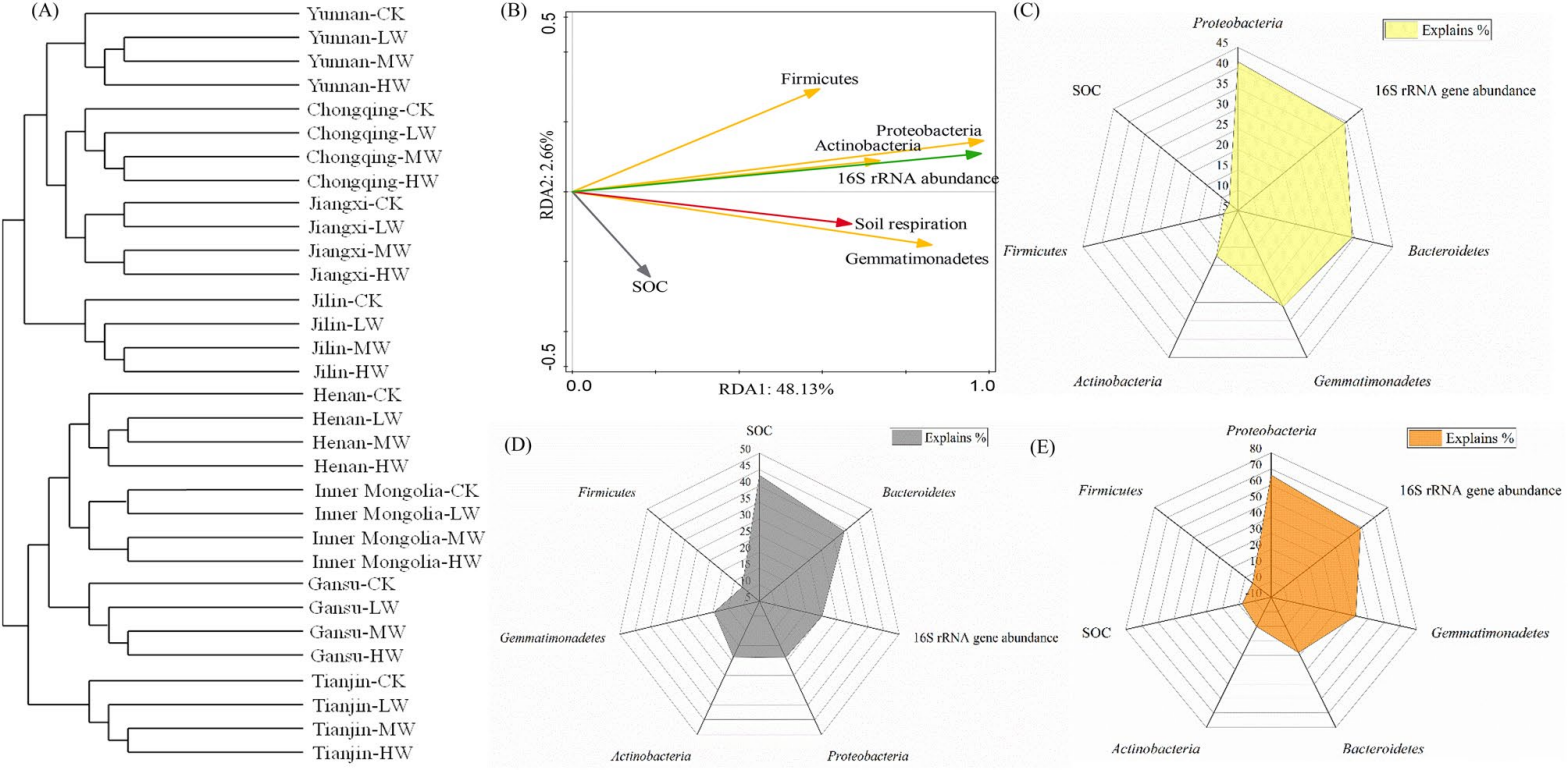
The unweighted pair group method with arithmetic mean of soil bacterial communities (**A**) and RDA analysis between soil respiration, soil organic carbon and the abundance of dominant carbon-relating bacteria in all soils (**B**,**C**), in acidic soils (**D**) and the alkaline soils (**E**) with different dosages of Cd-contamination and wheat straw addition.

In this study, carbon-relating bacteria were analyzed through 16S rRNA sequencing. After incubating with different dosages of Cd-contamination and WS amendment, *Proteobacteria*, *Actinobacteria*, *Firmicutes*, *Bacteroidetes* and *Gemmatimonadetes* were predominant phylum in the soils, averagely occupying 67.97–89.59% of the studied bacterial communities (Fig. 3B). According to the calculated results, the abundance of *Proteobacteria*, *Actinobacteria*, *Firmicutes*, *Bacteroidetes* and *Gemmatimonadetes* in the alkaline soils (1.52 × 10^11^ copies g^−1^, 3.91 × 10^10^ copies g^−1^, 2.84 × 10^10^ copies g^−1^, 1.83 × 10^10^ copies g^−1^, 2.02 × 10^10^ copies g^−1^) were higher than that in the acidic soils (9.22 × 10^10^ copies g^−1^ soil, 3.68 × 10^10^ copies g^−1^, 2.27 × 10^10^ copies g^−1^, 1.42 × 10^10^ copies g^−1^, 1.12 × 10^10^ copies g^−1^) (Fig. 4). In addition, compared with the CK treatment, different dosages of Cd-contamination decreased the abundance of dominated carbon-relating bacteria especially in the high dosages of Cd-contaminated soils (8.60–46.48%). Among all dominated phyla, different dosages of Cd-contamination weakly reduced the abundance of *Bacteroidetes* (8.60–14.22%), while largely decreased the abundance in the phylum *Proteobacteria*, *Actinobacteria*, *Firmicutes* and *Gemmatimonadetes* (18.62–46.48%). Furthermore, the decreased rate of *Bacteroidetes* in the acidic soils (3.92–8.93%) was lower than that in the alkaline soils (11.30–21.66%). Consistent with the concluded results presented in 16S rRNA gene bacterial structure, the eight soil samples could be grouped into two groups (Fig. 5A). The acidic soils with different dosages of Cd-contamination and wheat straw addition were obviously separated from the alkaline soils. In all types of soils, the CK soils with no Cd-contamination tended to be grouped firstly and then grouped with the other different dosages of Cd-contaminated soils.

### Correlation with cumulative C mineralization, TOC and bacterial communities

The correlation between cumulative C mineralization, soils organic carbon and the abundance of bacterial communities was evaluated by RDA analysis and correlation analysis (Fig. 5). In all soils with wheat straw addition and different dosages of Cd-contamination, *Proteobacteria* (r = 0.644, P < 0.01) largely explained the changes in cumulative C mineralization among all carbon-relating bacteria, followed by *Bacteroidetes* (r = 0.588, P < 0.01) and *Gemmatimonadetes* (r = 0.559, P < 0.01). In acidic soils, *Bacteroidetes* (r = 0.0.625, P < 0.01) had the largest explanation rate for explaining cumulative C mineralization. In alkaline soils, the larger explanation rate was observed in *Proteobacteria* (r = 0.814, P < 0.01) and *Gemmatimonadetes* (r = 0.651, P < 0.01) compared with other dominant bacteria. Additionally, the analyzed results showed there were no significant association between TOC concentration and the abundance of various dominant carbon-relating bacteria.

### Microbial functional predictions related with carbon metabolism

**Figure 6:**
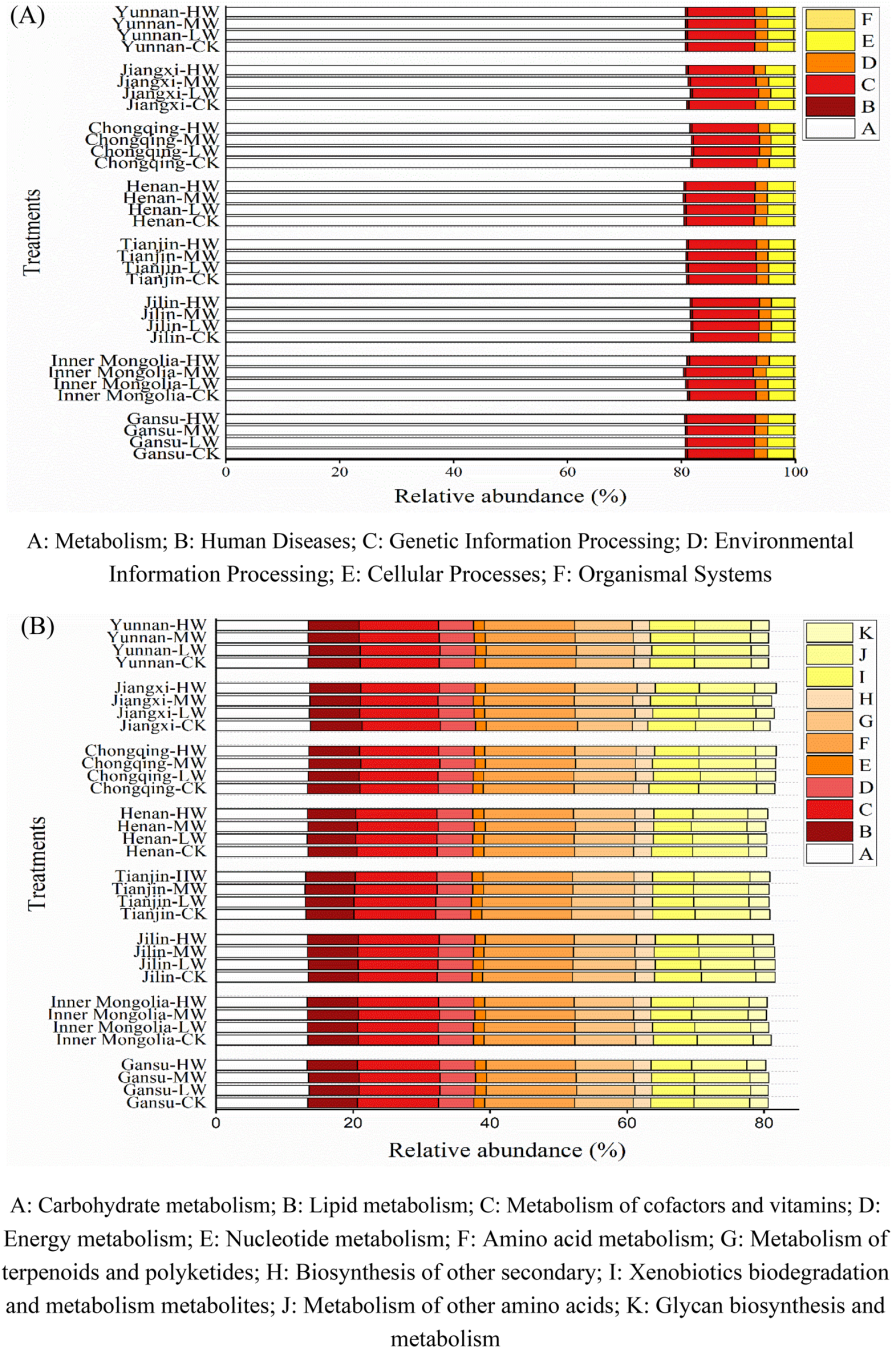
Changes in the microbial functional profiles obtained by phylogenetic investigation of communities by reconstruction of unobserved states (PICRUSt). (**A**) Biochemical metabolic pathways; (**B**) level 2 KEGG function predictions in terms of the relative abundances for the functions related to metabolism. (CK: 0 mg kg^−1^ Cd-contaminated soil with 5% WS addition; LW: 1 mg kg^−1^ Cd-contaminated soil with 5% WS addition; MW: 5 mg kg^−1^ Cd contaminated-soil with 5% WS addition HW: 10 mg kg^−1^ Cd contaminated-soil with 5% WS addition).

The impact of Cd dosages and soil types on the changes in microbial functions in all soils was conducted by PICRUSt functional predictions. As shown in Fig. 6, the predicted microbial sequences in all soils with Cd-contamination and wheat straw addition were distributed to six functions comprising metabolism (80.36–81.78%), genetic information processing (11.29–12.18%), environmental information processing (2.03–2.29%), cellular processes (3.88–4.82%), human diseases (0.30–0.46%), and organismal systems (0.26–0.33%). Metabolism was predominant among these microbial functional sequences, which occupied 11 pathways totally. Among all pathways of metabolism clusters, carbohydrate metabolism (12.99–13.65%) and amino acid metabolism (12.98–13.34%) were the main pathways. Compared with the CK treatment, the high dosages of Cd-contamination decreased the average abundance of genes correlated with amino acid metabolism and carbohydrate metabolism in all soils with wheat straw addition. But there was no regular difference between the CK treatment, medium-dose and low-dose of Cd-contaminated treatments. Additionally, higher average abundance in carbohydrate metabolism and amino acid metabolism was observed in the alkaline soils relative to that in the acidic soils.

## Discussion

### Impact of Cd-contamination on changes in soil carbon variations

The results of incubation experiments revealed that cadmium inhibit microbial activities and further restrict the TOC decomposition in different types of soil with wheat straw addition. The lowered CO_2_ emission was observed in the contaminated soil compared to the control, which could be used to confirm the above conclusion^18,24^. The different dosages of Cd-contamination greatly affected the abundance of bacterial communities. Correlation analysis instructed that soil bacterial abundance was positively associated with cumulative C mineralization, indicating the changes in soil bacterial abundance play important roles in cumulative C mineralization. Among different dosages of Cd-contaminated soils, high-dose Cd significantly decreased the bacterial 16S rRNA abundance and thus reduced cumulative C mineralization and enhanced TOC content in all types of soils with wheat straw addition. However, low-dose and medium-dose of Cd decreased the 16S rRNA gene abundance in all soils, while had no regular effect on cumulative C mineralization and TOC content. The similar results have been indicated by previous study of Chen et al.^24^. Lowered dosage of Cd-contamination (1 mg kg^−1^ and 5 mg kg^−1^) main reasonably explained the phenomenon. Most microorganisms can resist Cd toxicity through a variety of biochemical reactions including enzymatic oxidation, extracellular precipitation, intracellular complexation, and etc., which has been reported by Dong et al.^25^. *Proteobacteria*, *Actinobacteria*, *Firmicutes*, *Bacteroidetes* and *Gemmatimonadetes* were dominant carbon-relating bacteria have been found in substantial researches^26–28^. The outstanding functions of the above phyla are to mineralize cellulose, hemicellulose and lignin^29^. In this study, the correlation analysis showed the abundance of dominant carbon-relating bacteria was associated with cumulative C mineralization, but had no relationship with TOC content. The results indicated that changes in carbon-relating bacteria directly affect cumulative C mineralization, but indirectly influence the TOC content. Among all carbon-relating phyla, *Proteobacteria*, *Bacteroidetes* and *Gemmatimonadetes* played an important role in cumulative C mineralization under all soils with Cd-contamination and wheat straw addition. The species of *Proteobacteria* was the most dominant phylum in all soils and have high tolerance to Cd has been found^30,31^. Additionally, *Bacteroidetes* and *Gemmatimonadetes* are dominated bacterial species in Cd-contaminated soils has been also reported by Li et al.^32^. An et al.^33^ had indicated *Gemmatimonadetes* are highly tolerant to Cd stress. The abundance of *Proteobacteria*, *Bacteroidetes* and *Gemmatimonadetes* was greatly influenced by higher dosages of Cd-contamination. The results showed that the composition of bacterial communities in different soils exist significant differences, which may be attributed to the dynamics of soil physicochemical properties.

### Effect of soil types on changes in cumulative C mineralization

Soil types also influence the abundance and structure of bacteria communities and TOC content. Compared with that in the alkaline soils, the abundance of bacterial 16S rRNA gene and dominant carbon-relating bacteria were lower in the acidic soils. In alkaline soil, it can be seen that increasing Cd concentration negatively impact on cumulative C mineralization. This pattern was consistent with the findings of Yeates et al.^34^ and Ohya et al.^35^. Furthermore, the lower cumulative C mineralization and higher TOC content could be observed in the acidic soils relative to that in the alkaline soils. The findings demonstrated that cadmium inhibit microbial activities and further restrict the soil organic carbon decomposition in acidic soils with wheat straw addition compared with that in the alkaline soils. The difference in the effects of Cd concentrations on cumulative C mineralization in various types of soils may be related to the microbial toxicity of cadmium^36^. This may be attributed to the tendency of Cd to be mobilized under acidic conditions. Previous researches have indicated that soil pH was one of the most important parameters influencing Cd fractions in studied soil, which was negatively associated with available Cd content through increasing competition for negative ions surfaces between Cd^2+^ and H^+^^37,38^. The results indicated that pH is an important factor to influence the decomposition and mineralization of organic carbon through effecting microbial activities^39,40^. The decomposition rate of organic carbon increased with the decrease of pH, which was owing to the strong acidic environment inhibited microbial activities and decreased the mineralization of organic carbon. The highest cumulative C mineralization in Inner Mongolia soils and the lowest value in Chongqing soils were highly consistent with the pH in these two soils, which also confirmed this point. The cumulative C mineralization was highly related to microbial activities both in acidic soils and in the alkaline soils. Creamer et al.^41^ indicated that bacterial communities have a positively effects on soil carbon mineralization and soil carbon respiration. In this study, the results of 16S rRNA gene sequencing showed *Bacteroidetes* contributed more to cumulative C mineralization in the acidic soils. *Bacteroidetes* have a high adaptability to acidic environment, which can be confirmed by previous research results that it can convert lignocellulose into small molecule fatty acid chain, thus decreasing the pH of the environment^42^. However, *Proteobacteria* and *Gemmatimonadetes* had the highest correlation with cumulative C mineralization in the alkaline soils. *Proteobacteria* and *Gemmatimonadetes* were dominant bacterial species in the Cd-contaminated alkaline soils^43^. As the obtained results, *Bacteroidetes*, *Proteobacteria* and *Gemmatimondetes* were closely correlated with pH, suggesting that the growth of these bacteria were likely to influence or be influenced by changes of pH in Cd-contaminated soils with wheat straw addition.

### The effect of Cd-contamination needs further field verification

The results of incubation experiment demonstrated that Cd contamination further inhibits the cumulative C mineralization through limiting microbial activities, which is more beneficial to enhance TOC content in soil. Furthermore, the cumulative C mineralization was largely affected in the soil with various pH levels. Therefore, the addition of wheat straw is an effective measure for enhancing TOC content in acidic Cd-contaminated soil. Six predicted functional sequences including metabolism, genetic information processing, environmental information processing, cellular processes, human diseases, and organismal systems. The results were similar with the findings reported by Duan et al.^44^. Metabolism contributed more to the decomposition of organic carbon in soil has been previously reported by Zhou et al.^45^. Microbial functional predictions related with carbon metabolism were mainly belonged to carbohydrate metabolism and amino acid metabolism. Carbohydrate metabolism was associated with the decomposition of cellulose and hemicellulose with the presence of microorganisms^46^. Amino acid metabolism could produce more amino acid and humic acid under the activity of microorganisms^47^. The high-dose of Cd-contamination decreased the abundances of carbohydrate metabolism and amino acid metabolism relative to the CK treatment, indicating that high-dose of Cd-contamination promoted the inhibition of microbially mediated organic carbon decomposition. Additionally, it was also concluded that Cd-contamination largely inhibited organic carbon degradation in the acid soils relative to that in the alkaline soils according to the distribution in different types of soils. However, whether cadmium has a strong effect on the accumulation of soil carbon in the field requires further field examination. Ohya et al.^35^ found that the activity of microorganisms was not only limited with heavy contamination but also constrained by laboratory condition. Therefore, the response of microbially mediated organic carbon decomposition to heavy metal contamination needs further field verification. Furthermore, the difference in the relative contribution of bacteria communities to soil carbon changes in the acidic soils and in the alkaline soils need to be further tested and validated by introducing more soil types.

## Conclusion

Cd-contamination inhibited the microbially mediated organic carbon decomposition through decreasing bacterial abundance and limiting bacterial activities, which is more beneficial to enhance TOC content in soil. In particular, high dosage of cadmium contamination has a stronger inhibiting effect on cumulative C mineralization. Inhibition of microbially mediated organic carbon decomposition is affected by soil types. Cadmium inhibited bacterial activities and further restricted the TOC decomposition in acidic soils with wheat straw addition compared with that in the alkaline soils. *Proteobacteria* and *Gemmatimonadetes* were abundant in alkaline Cd-contaminated soils with wheat straw addition, while *Bacteroidetes* dominated cumulative C mineralization in acidic Cd-contamination soils. These findings have implications for guiding field verification to understand the mechanisms of soil carbon dynamics in the heavy metal contaminated soils with wheat straw returning.

### Supplementary Information

Supplementary Information.

## Data Availability

All data generated or analysed during this study are included in this published article (and its supplementary information files).

## References

[1] Conrad R, Klose M, Yuan Q, Lu YH, Chidthaisong A (2012). Stable carbon isotope fractionation, carbon flux partitioning and priming effects in anoxic soils during methanogenic degradation of straw and soil organic carbon. Soil Biol. Biochem..

[2] Muhammad W, Vaughan SM, Dalal RC, Menzies NW (2011). Crop residues and fertilizer nitrogen influence residue decomposition and nitrous oxide emission from a Vertisol. Biol. Fertil. Soils.

[3] Garcia-Pausas J, Paterson E (2011). Microbial community abundance and structureare determinants of soil organic carbon mineralization in the presence of labile carbon. Soil Biol. Biochem..

[4] Zhang HJ, Ding WX, Yu HY, He XH (2015). Linking organic carbon accumulation to microbial community dynamics in a sandy loam soil: Result of 20 years compost and inorganic fertilizers repeated application experiment. Biol. Fertil. Soils.

[5] Wang C, Li W, Guo M, Ji J (2017). Ecological risk assessment on heavy metals in soils: Use of soil diffuse reflectance mid-infrared Fourier-transform spectroscopy. Sci. Rep..

[6] Zwolak A, Sarzyńska M, Szpyrka E, Stawarczyk K (2019). Sources of soil pollution by heavy metals and their accumulation in vegetables: A review. Water Air Soil Pollut..

[7] Gu J, Zhou Q, Wang X (2003). Reused path of heavy metal pollution in soils and its research advance. J. Basic Sci. Eng..

[8] Kandeler E, Tscherko D, Bruce KD, Stemmer M, Hobbs PJ, Bardgett RD (2000). Structure and function of the soil microbial community in microhabitats of a heavy metal polluted soil. Biol. Fertil. Soils.

[9] Walker C, Goodyear C, Anderson D, Titball RW (2000). Identification of arsenic-resistant bacteria in the soil of a former munitions factory at Löcknitz, Germany. Land Contam. Reclam..

[10] Shen GQ, Cao LK, Lu YT, Hong JB (2005). Influence of phenanthrene on cadmium toxicity to soil enzymes and microbial growth. Environ. Sci. Pollut. Res..

[11] McGrath SP, Zhao FJ, Lombi E (2001). Plant and rhizosphere processes involved in phytoremediation of metal-contaminated soils. Plant Soil.

[12] Chaerun SK, Pangesti NPD, Toyota K, Whitman WB (2011). Changes in microbial functional diversity and activity in paddy soils irrigated with industrial wastewaters in Bandung, west Java province, Indonesia. Water Air Soil Pollut..

[13] Cui YS, Fu J, Chen XC (2011). Speciation and bioaccessibility of lead and cadmium in soil treated with metal-enriched Indian mustard leaves. J. Environ. Sci..

[14] Liang J, Yang Z, Tang L, Zeng G, Yu M, Li X, Wu H, Qian Y, Li X, Luo Y (2017). Changes in heavy metal mobility and availability from contaminated wetland soil remediated with combined biochar-compost. Chemosphere.

[15] Jayalath N, Fitzpatrick RW, Mosley L, Marschner P (2016). Type of organic carbon amendment influences pH changes in acid sulfate soils in flooded and dry conditions. J. Soil Sediment..

[16] Darma A, Yang JJ, Feng Y, Xia X, Zandi PM, Sani A, Bloem E, Ibrahim S (2023). The impact of maize straw incorporation on arsenic and cadmium availability, transformation and microbial communities in alkaline-contaminated soils. J. Environ. Manag..

[17] Fließbach A, Martens R, Reber HH (1994). Soil microbial biomass and microbial activity in soils treated with heavy metal contaminated sewage sludge. Soil Biol. Biochem..

[18] Chen YP, Liu Q, Liu YJ, Jia FA, He XH (2014). Responses of soil microbial activity to cadmium pollution and elevated CO_2_. Sci. Rep..

[19] Yuan CL, Zhang LM, Wang JT, Hu HW, Shen JP, Cao P, He JZ (2019). Distributions and environmental drivers of archaea and bacteria in paddy soils. J. Soil Sediment..

[20] Yang YJ, Wang H, Li CJ, Liu HX, Fang XH, Wu MY, Lv JL (2024). Identification of the soil physicochemical and bacterial indicators for soil organic carbon and nitrogen transformation under the wheat straw returning. PLoS ONE.

[21] Yang YJ, Liu HX, Dai YC, Tian HX, Zhou W, Lv JL (2020). Soil organic carbon transformation and dynamics of microorganisms under different organic amendments. Sci. Total Environ..

[22] Bao HY, Wang JF, Zhang H, Li J, Li H, Wu FY (2020). Effects of biochar and organic substrates on biodegradation of polycyclic aromatic hydrocarbons and microbial community structure in PAHs-contaminated soils. J. Hazard. Mater..

[23] Bao SD (2000). Soil and Agricultural Chemistry Analysis.

[24] Zhang C, Nie S, Liang J, Zeng G, Wu H, Hua S, Xiang H (2016). Effects of heavy metals and soil physicochemical properties on wetland soil microbial biomass and bacterial community structure. Sci. Total Environ..

[25] Dong J, Mao WH, Zhang GP, Wu FB, Cai Y (2007). Root excretion and plant tolerance to cadmium toxicity—A review. Plant Soil Environ. UZPI (Czech Republic).

[26] Wang Q, Garrity GM, Tiedje JM, Cole JR (2007). Naive Bayesian classifier for rapid assignment of rRNA sequences into the new bacterial taxonomy. Appl. Environ. Microbiol..

[27] Awasthi MK, Chen HY, Wang Q, Liu T, Duan YM, Awasthi SK, Ren XN, Tu ZN, Li J, Zhao JC, Zhang ZQ (2018). Succession of bacteria diversity in the poultry manure composted mixed with clay: Studies upon its dynamics and associations with physicochemical and gaseous parameters. Bioresour. Technol..

[28] Han SI, Lee JC, Lee HJ, Whang KS (2013). *Planifilum composti* sp. nov., a thermophile isolated from compost. Int. J. Syst. Evol. Microbiol..

[29] Blanc M, Marilley L, Beffa T, Aragno M (2010). Thermophilic bacterial communities in hot composts as revealed by most probable number counts and molecular (16S rDNA) methods. FEMS Microbiol. Ecol..

[30] Kevin F, Philip R, Chris F, Moore JN, Gannon JE, Holben WE (2003). Differences in hyporheic-zone microbial community structure along a heavy-metal contamination gradient. Appl. Environ. Microbiol..

[31] Lorenz N, Hintemann T, Kramarewa T, Katayama A, Kandeler E (2006). Response of microbial activity and microbial community composition in soils to long-term arsenic and cadmium exposure. Soil Biol. Biochem..

[32] Li X, Meng D, Li J, Yin H, Liu H, Liu X (2017). Response of soil microbial communities and microbial interactions to long-term heavy metal contamination. Environ. Pollut..

[33] An F, Li H, Diao Z, Lv J (2018). The soil bacterial community in cropland is vulnerable to Cd contamination in winter rather than in summer. Environ. Sci. Pollut. Res..

[34] Yeates GW, Orchard VA, Speir TW, Hunt JL, Hermans MCC (1994). Impact of pasture contamination by copper, chromium, arsenic timber preservative on soil biological activity. Biol. Fertil. Soils.

[35] Ohya H, Fujiwara S, Komai Y, Yamaguchi M (1988). Microbial biomass and activity in urban soils contaminated with Zn and Pb. Biol. Fertil. Soils.

[36] Rajapaksha RMCP, Tobor-Kapłon MA, Bååth E (2004). Metal toxicity affects fungal and bacterial activities in soil differently. Appl. Environ. Microbiol..

[37] Shahid M, Dumat C, Khalid S, Niazi NK, Antunes PMC (2016). Cadmium bioavailability, uptake, toxicity and detoxification in soil-plant system. Rev. Environ. Contam. Toxicol..

[38] Ardestani MM, van Gestel CAM (2013). Using a toxicokinetics approach to explain the effect of soil pH on cadmium bioavailability to *Folsomia candida*. Environ. Pollut..

[39] Pietri JA, Brookes PC (2008). Relationshipsbetween soil pH and microbial properties in a UK arable soil. Soil Biol. Biochem..

[40] Lauber CL, Hamady M, Knight R, Fierer N (2009). Pyrosequencing-based assessment of soil pH as a predictor of soil bacterial community structure at the continental scale. Appl. Environ. Microb..

[41] Creamer CA, de Menezes AB, Krull ES, Sanderman J, Newton-Walters R, Farrell M (2015). Microbial community structure mediates response of soil C decomposition to litter addition and warming. Soil Biol. Biochem..

[42] Peng S, Li HJ, Song D, Lin XG, Wang YM (2018). Influence of zeolite and superphosphate as additives on antibiotic resistance genes and bacterial communities during factory-scale chicken manure composting. Bioresour. Technol..

[43] An MJ, Wei CZ, Wang KY, Fan H, Wang XL, Chang DD (2021). Effects of polymer modifiers on the bacterial communities in cadmium-contaminated alkaline soil. Appl. Soil Ecol..

[44] Duan ML, Zhang YH, Zhou BB, Qin ZL, Wu JH, Wang QJ, Yin YN (2020). Effects of Bacillus subtilis on carbon components and microbial functional metabolism during cow manure-straw composting. Bioresour. Technol..

[45] Zhou G, Xu X, Qiu X, Zhang J (2019). Biochar influences the succession of microbial communities and the metabolic functions during rice straw composting with pig manure. Bioresour. Technol..

[46] Toledo M, Gutiérrez MC, Siles JA, García-Olmo J, Martín MA (2017). Chemometric analysis and NIR spectroscopy to evaluate odorous impact during the composting of different raw materials. J. Clean Prod..

[47] Wei H, Wang L, Hassan M, Xie B (2018). Succession of the functional microbial communities and the metabolic functions in maize straw composting process. Bioresour. Technol..

