# Supplementary material for: Inhibition of microbially mediated total organic carbon decomposition in different types of cadmium contaminated soils with wheat straw addition
Source: Sci Rep. 2024 Jul 2;14:15114. doi: 10.1038/s41598-024-64267-2 (PMC11219759; doi:10.1038/s41598-024-64267-2)
Supplement: Supplementary file 1 — Supplementary Information. [file 41598_2024_64267_MOESM1_ESM.docx]

**SUPPORTING INFORMATION**

**Inhibition of microbially mediated organic matter decomposition in different types of cadmium contaminated soils with wheat straw addition**

Chengjuan Li^b,1^, Hui Wang^b,1^, Yajun Yang^a,^*, Hexiang Liu^a^, Xianhui Fang^a^, Yaohui Zhang^a^, Jialong Lv^a^

*^a^State Key Laboratory of Soil Erosion and Dryland Farming on the Loess Plateau, Institute of soil and water conservation Chinese Academy of Sciences & College of Natural Resources and Environment, Northwest A&F University, Yangling, Shaanxi Province 712100, PR China*

*^b^Xianyang Soil and Fertilizer Workstation, Xianyang, Shaanxi Province 712000, PR China*

*2.1. Soil and wheat straw used for incubation*

**Table S1** The basic properties of soils before the incubation

| Soils | Locations | Properties | | | | | | | | |
| --- | --- | --- | --- | --- | --- | --- | --- | --- | --- | --- |
|  |  | Clay (%) | CEC (cmol kg^-1^) | pH | CaCO_3_ (g kg^-1^) | TOC (g kg^-1^) | TN (g kg^-1^) | AP  (mg kg^-1^) | AK  (mg kg^-1^) | Background Cd (mg kg^-1^) |
| Inner Mongolia | 41°33' N, 110°01' E | 10.51 | 11.61 | 8.80 | 11.51 | 9.45 | 1.00 | 13.89 | 119.6 | 0.22 |
| Gansu | 38°52' N, 100°26' E | 6.66 | 11.23 | 8.37 | 38.51 | 11.18 | 0.93 | 26.30 | 135.7 | 0.21 |
| Henan | 35°00' N, 113°41' E | 18.18 | 16.01 | 8.07 | 27.50 | 10.32 | 0.99 | 60.00 | 79.15 | 0.23 |
| Tianjin | 38°45' N, 117°06' E | 7.59 | 24.67 | 8.29 | 53.57 | 12.77 | 1.45 | 59.91 | 210.3 | 0.22 |
| Chongqing | 29°48' N, 106°24' E | 24.96 | 21.34 | 5.74 | 0.00 | 10.14 | 0.94 | 46.60 | 68.05 | 0.20 |
| Jilin | 43°31' N, 124°48' E | 30.18 | 31.11 | 6.82 | 0.00 | 19.05 | 1.65 | 82.88 | 127.0 | 0.14 |
| Jiangxi | 28°12' N, 116°56' E | 36.51 | 8.70 | 6.01 | 0.00 | 6.78 | 0.65 | 30.34 | 138.2 | 0.18 |
| Yunnan | 24°52' N, 102°49' E | 27.52 | 11.10 | 5.92 | 0.00 | 19.87 | 1.68 | 54.53 | 170.0 | 0.30 |

CEC: cation exchange capacity; TOC: total organic carbon; TN: total nitrogen; AP: available phosphorus; AK: available potassium

**S. The details of qPCR reaction system and amplification conditions**

The qPCR reaction system comprised 1 μL DNA template, 0.5 μL 10 μM forward and reverse primers (Beijing Auwigene Tech, Ltd, China), 12.5 μL 2×MltraSYBR Mixture, and 10.5 μL of sterilized double distilled H_2_O.

The qPCR amplification reaction was as follows: (1) initial denaturation for 5 min at 94°C; (2) 40 cycles at: 94°C for 30 s, annealing for 30 s at 60°C, and then extension at 72°C for 1 min. In order to eliminate the effects of PCR inhibitors on qPCR in the DNA extracts, the DNA template used for qPCR was diluted 10 times. The presence of inhibitory compounds in the extracted DNA was checked by qPCR using serially diluted samples. Melting curve analysis was used to detect nonspecific amplification. Each gene was quantified in triplicate using a standard curve and a negative control. The gene copy numbers in the samples were calculated using the external standard curve method. The abundances of genes were calculated as: copy number of gene/copy number of 16S rDNA.
